# Mucinous Tubular and Spindle Cell Carcinoma: Case Report and Literature Review

**DOI:** 10.15586/jkc.v12i1.354

**Published:** 2025-02-25

**Authors:** SL Tsang, SS Hsu, Cheung AN, SHB Ho, ATL Ng

**Affiliations:** 1 Division of Urology, Department of Surgery, The University of Hong Kong, Queen Mary Hospital, Hong Kong;; 2 Department of Pathology, The University of Hong Kong, Queen Mary Hospital, Hong Kong

**Keywords:** renal cell carcinoma, kidney neoplasms, mucinous tubular and spindle cell carcinoma

## Abstract

Mucinous tubular and spindle cell carcinoma (MTSCC) is a rare type of renal cell carcinoma (RCC) recognized as an independent entity in the latest WHO (World Health Organization) classification. We here report a case of a 51-year-old female patient with MTSCC, who presented with abdominal pain and left lower pole kidney lesion on the computed tomography scan. A robotic-assisted laparoscopic partial nephrectomy was performed. The diagnosis was confirmed on histopathological examination. MTSCC is rare and generally indolent. Either partial or radical nephrectomy is usually curative. The prognosis is usually favorable. However, occasionally, MTSCC could demonstrate aggressive features requiring systemic therapy. There are also several mimickers of MTSCC, which carry different prognostic and treatment profiles. Histological, immunohistochemical, and molecular genetic profile are useful in diagnosing the disease.

## Introduction

Mucinous tubular and spindle cell carcinoma (MTSCC) is a rare type of renal cell carcinoma (RCC) recognized as an independent entity in the latest 2022 WHO (World Health Organization) classification (1). MTSCC is generally indolent in behavior and excision is usually the treatment of choice. Nevertheless, there are some mimickers that demonstrate different prognostic and treatment implications. Vigilant histopathological examination is crucial to guide the management. Literatures of MTSCC are predominantly case reports and series. In this paper, we present a case of MTSCC and a comprehensive literature review.

## Case Report

**Figure 1:**
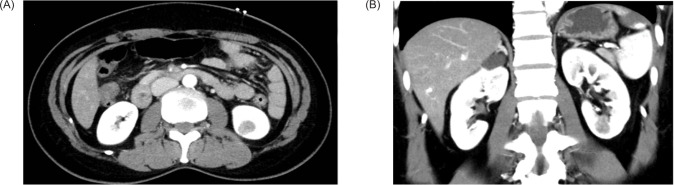
Contrast-enhanced computed tomography images. (A) axial image, (B) coronal image.

A 51-year-old lady presented to our institute with abdominal pain. Physical examination was unremarkable. Ultrasound of the abdomen showed a 1.6 cm hyperechoic mass at the lower pole of the left kidney. A computed tomography (CT) scan was thus performed, which showed a 1.7 × 1.4 × 1.7 cm hypodense contrast-enhanced lesion in the lower pole of the left kidney (Figure 1); the mass was mainly endophytic. There was no invasion to the perinephric fat and the renal vein was patent. The RENAL nephrometry score was 7. A robotic-assisted laparoscopic left partial nephrectomy was performed with the left lower pole branch selective clamping. The total warm ischemic time was 25 minutes. There was no complication.

**Figure 2:**
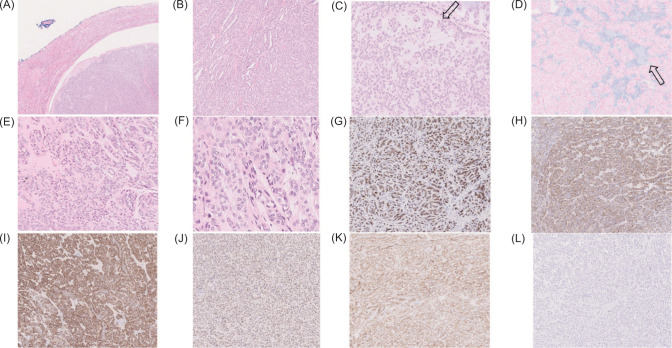
Histopathology and immunochemistry images. (A) The nodular tumor is partly covered by a thin fibrous capsule and partly by renal tissue. (B) Elongated tubular structures and cord-like growth. (C) The stroma is myxoid with areas of extracellular mucin. (D) The mucin is highlighted by Alcian Blue staining. (E and F) Spindle cells are seen adjacent to cuboidal tumor cells. (G to K) The tumor cells are positive for EMA (G), AMACR (H), CK7 (I), PAX8 (J), and CD56 (K). (L) MIB1 is generally less than 1%.

The gross examination of specimen revealed a pinkish nodule measuring 2.2 × 2 × 1.5 cm and weighing 3.8 g. No necrosis was noted. Microscopically, the tumor was covered by a thin fibrous capsule partly surrounded by a thin rim of renal tissue (Figure 2A). The tumor consisted of closely packed tubules with scattered long tubular structures (Figure 2B). There was myxoid stroma and the extracellular mucin areas were highlighted by Alcian Blue staining (Figure 2C and 2D). The cuboidal tumor cells had moderate amount of pale to light eosinophilic cytoplasm with transition to spindle cells (Figure 2E and 2F). Mitotic activity was inconspicuous, and necrosis was not found. Immunohistochemically, it showed positivity for EMA (epithelial membrane antigen) (Figure 2G), AMACR (alpha-methylacyl-CoA racemase) (Figure 2H), CK7 (cytokeratin 7) (Figure 2I), PAX8 (Figure 2J), and CD56 (Figure 2K). MIB-1 proliferation index showed a rate of less than 1% (Figure 2L). The diagnosis of MTSCC was confirmed. The TNM (tumor, node, metastasis) stage was pT1a; no adjuvant treatment was given; and a surveillance scan was arranged.

## Discussion

MTSCC is a rare type of RCC, first recognized as a distinct entity in the 2004 WHO guideline. It was previously classified under different subtypes of RCC, including low-grade collecting duct carcinoma, RCC with unusual differentiation, low-grade tubular mucinous renal neoplasm, and so on and so forth. It primarily impacts females (3–4:^1^) and has a broad age range of presentation, extending from 13 to 82 years (mean age 53 years) (2, 3). Fewer than 50 cases were reported in the Chinese population, and never reported in Hong Kong. Most MTSCC are found as incidental findings. Some present with symptoms such as flank pain, abdominal mass, and hematuria. Radiologically, it shows iso- or slight hypoattenuation on CT. On magnetic resonance imaging, it usually demonstrates mild hypointensity with focal hyperintensity on the T2 phase, diffusion restriction on DWI (diffusion-weighted imaging), and multiple patchy enhancement in the corticomedullary phase (4).

Macroscopic examination commonly shows a well-defined tumor located in the renal cortex, where the surface is usually shiny and mucoid with homogenous color and firm in consistency (2). Microscopically, MTSCC is usually characterized by a combination of tubular and spindle cells separated by various amounts of mucinous stroma. Tubules are usually arranged parallelly and packed. Transitions between tubular and spindle cells are often seen. Variations in pattern such as mucin-poor, spindle cell-dominant, and high nuclear grade presentations were reported (5). Necrosis and mitoses are rare. A tumor with aggressive behavior usually exhibits atypical histological features such as high-grade transformation and sarcomatoid transformation (2). High-grade transformation of MTSCC demonstrates features like necrosis, high-grade nuclei, increased mitosis, vascular invasion, and solid or infiltrative growth (6). Sarcomatoid transformation typically shows an expansile growth of high-grade spindle cells (7). Immunohistochemically, MTSCC consistently show positivity for EMA (95%), AMACR (93%), and CK7 (81%) (8). Variable expressions are shown in HMWK (high molecular weight kininogen) (15%), CD10 (15%), RCC Ma (7%), and c-kit (5%).

The immunohistochemical profile (above) shows similarity to that of papillary RCC (PRCC) (1). Therefore, PRCC with sarcomatoid differentiation, which has poorer outcomes, is sometimes regarded as a mimicker of MTSCC. Yet, MTSCC has a distinct molecular genetic profile. FISH (fluorescence in situ hybridization)-based studies showed that MTSCC commonly exhibit multiple chromosomal losses involving chromosomes 1, 4, 6, 8, 9, 13, 14, 15, and 22. Clinically aggressive MTSCC may also show CDKN2A/2B (9p) deletion and additional complex genomic alternations (6, 9). Other differential diagnoses include sarcomatoid RCC, mesenchymal neoplasms such as smooth muscle tumors (SMTs), and inflammatory myofibroblastic tumor (IMT) (10). Sarcomatoid RCC usually demonstrates pleomorphic and hyperchromatic nuclei and increased mitotic activity and necrosis (11). SMT and IMT usually exhibit more elongated nuclei and clear fascicular arrangement, and strongly positive for SMA (alpha smooth muscle actin) (Table 1) (12).

**Table 1:** Comparison of MTSCC with other relevant differential diagnoses.

	Mucinous Tubular and Spindle Cell Carcinoma (MTSCC)	Papillary Renal Cell Carcinoma (PRCC)	Sarcomatoid Renal Cell Carcinoma (SRCC)	Smooth Muscle Tumor (SMT)	Inflammatory Myofibroblastic Tumor (IMT)
**Histopathology**	Tightly packed tubules lined by low-grade cuboidal cells merging with bland spindle cells in myxoid stroma containing mucin	Papillary or tubular architecture lined by cuboidal cells with scant or lightly basophilic cytoplasm	Spindle cells with sarcomatoid features, often with rhabdoid or pleomorphic cells	Spindle cells with smooth muscle differentiation Malignant SMT demonstrates nuclear atypia, hyperchromasia, pleomorphism, and high mitotic rate	Spindled myofibroblasts, fibroblasts, and inflammatory cells
**Immunohistochemistry**	Positive for PAX8, CK7, and AMACR	Positive for CK7, AMACR, vimentin, and CD10	Often positive for vimentin and cytokeratins	Positive for SMA, desmin, and caldesmon	Positive for SMA, desmin, vimentin, and ALK (~50%)
**Diagnostic molecular feature**	Multiple chromosomal loss VSTM2A RNA expression	Limited use Trisomy and tetrasomy of chromosomes 7 and 17 and loss of Y chromosomes MET gene mutations often associated with low-grade PRCC	Often associated with TP53 mutations and chromosomal instability	No specific genetic features	ALK rearrangements may be found
**Prognosis**	Generally favorable, with low metastatic potential	Intermediate prognosis, with metastatic potential	Poor prognosis, with high metastatic potential	Benign, with rare cases of malignant transformation	Benign, with rare cases of malignant transformation

MTSCC is usually considered a low-grade malignancy exhibiting an indolent course. Tumors are commonly of low stage (T1, T2) and are amenable to partial or radical nephrectomy. Few case reports and series showed recurrence, regional lymph node, and distant metastasis, usually associated with high-grade features (3). Yet, rare cases illustrated that MTSCC with classical morphology were also capable of metastatic behavior (13). The mainstay treatment for MTSCC remains surgical excision, either partial or radical nephrectomy. Although recurrence is unlikely, a close follow-up with surveillance scan is recommended. For metastatic MTSCC, there are currently no established guidelines or trials regarding systemic treatment. There are case reports showing long-lived response with the of use of sunitinib as first-line treatment (3). There are also case reports documenting the use of nivolumab or the combination of nivolumab plus ipilimumab (14).

The functional and oncological outcomes of MTSCC after partial nephrectomy is generally favorable—with preserved renal function and low recurrence rate. Although recurrence after partial nephrectomy is infrequent, early diagnosis is crucial as surgery is the most effective treatment. Besides, should there be recurrences, it usually occurs late (median 5–6 years) (15). Therefore, surveillance with imaging is recommended. The current European Association of Urology (EAU) guidelines advocate for a risk-stratified surveillance approach (16). For example, for the patient in this case report (with pT1a low-grade non-clear cell RCC), CT should be performed at 6, 18, and 30 months, followed by imaging every two years thereafter. Imaging for oncological surveillance may be discontinued after three years, taking into account the patient’s comorbidities, age, life expectancy, and preference.

## Conclusion

MTSCC is a type of rare and generally indolent renal tumor. Patients are often asymptomatic and tumors are found incidentally. The prognosis is usually favorable. Either partial or radical nephrectomy is usually curative. However, occasionally, MTSCC could demonstrate aggressive features. And there is a small chance of metastasis, requiring systemic therapy. There are also several mimickers of MTSCC, which carry different prognostic and treatment profiles. Clinicians and pathologists have to be vigilant when diagnosing MTSCC.
